# Gonadotropin inhibitory hormone downregulates steroid hormone secretion and genes expressions in duck granulosa cells

**DOI:** 10.1590/1984-3143-AR2021-0036

**Published:** 2021-07-19

**Authors:** Shijian Chen, Wenjun Liu, Chen Yang, Xiujin Li, Xu Shen, Danli Jiang, Yunmao Huang, Yunbo Tian

**Affiliations:** 1 Zhongkai University of Agriculture and Engineering, Guangdong Guangzhou, China; 2 Guangdong Province Key Laboratory of Waterfowl Healthy Breeding, Guangdong Guangzhou, China

**Keywords:** Gonadotropin-inhibitory hormone (GnIH), steroid synthesis, gene expression, reproduction, granulosa cell

## Abstract

The mechanisms by which GnIH regulates the steroid synthesis pathway in duck granulosa cells remain poorly understood. In this study, we measured steroid hormone secretion by ELISA and reproduction-associated gene expression by quantitative real-time Polymerase Chain Reaction (qPCR) in duck granulosa cells treated with different concentrations of GnIH (0, 0.1, 1, 10, and 100 ng/mL) for 24 h. The genome-wide expression profiles of GnIH-treated cells (0 and 10 ng/mL) were evaluated by high-throughput RNA sequencing. Compared with untreated cells, the secretion of the steroid hormones E2, E1, P4, and T was downregulated, with that of E1 and P4 reaching statistical significance (*P*<0.05); in contrast, the secretion of ACV and INH was significantly upregulated (*P*<0.05) after treatment with 10 and 100 ng/mL GnIH. The expression of encoding steroidogenic proteins and enzymes genes (*STAR*, *CYP11A1*, *CYP17A1*, *CYP19A1*, and *3-β-HSD*) and encoding gonadotropin receptors genes (*FSHR*, *LHR*) were significantly declined (*P*<0.05) in the 10 and 100 ng/mL GnIH treatments. Transcriptome sequencing identified 348 differentially expressed genes (DEGs), including 253 upregulated and 95 downregulated genes. The DEGs were mainly involved in cell growth and death, immune response, and steroid biosynthesis pathways. We identified four novel DEGs (*MROH5*, *LOC113840576*, *SDR42E1*, and *LOC113841457*) with key roles in the regulation of steroid hormone biosynthesis. Our study revealed changes in gonadal steroid hormone secretion and steroid biosynthesis pathway-related gene expression in duck granulosa cells under the inhibitory effect of GnIH. These data contribute to our understanding of the molecular and genetic mechanisms underlying reproduction in ducks.

## Introduction

In 2000, a hypothalamic neuropeptide isolated from the brain of Japanese quail, named gonadotropin inhibitory hormone (GnIH), was shown to inhibit the release of a gonadotropin (Tsutsui et al., 2000). GnIH plays an essential role in regulating animal reproduction and has inhibitory effects on reproductive regulation and gonadotropin expression. It can directly or indirectly inhibit the activity of gonadotropin-releasing hormone GnRH-expressing neurons in the hypothalamus to regulate the secretion and synthesis of pituitary gonadotropin (Ubuka et al., 2012). The addition of GnIH to cultures of diced pituitary glands from adult cockerels results in a significant decrease in the secretion levels of follicle-stimulating hormone (FSH) and luteinizing hormone (LH) (Ciccone et al., 2004).

GPR147 and GPR74 have been shown to act as G protein-coupled receptors for GnIH in gonadotropic cells and gonads (Yin et al., 2005). Previous studies have indicated that GnIH and its receptors are also expressed in follicle granulosa cells (Ubuka et al., 2013), suggesting that GnIH may have an autocrine/paracrine regulatory role in gonadal steroid production and germ cell maturation (Bentley et al., 2008; Ubuka et al., 2016). Under the stimulation of FSH and LH, granulosa cells synthesize estradiol and progesterone to regulate follicle maturation and ovulation. Previous studies have shown that GnIH reduces ovarian activity, inhibits follicular development, and also reduces the expression of the proliferation-related proteins ERK1/2, cyclin-B1, and PCNA in porcine ovarian granulosa cells (Singh et al., 2011a; Li et al., 2013). This suggests that GnIH not only acts on the hypothalamic–pituitary–gonadal (HPG) axis but also regulates ovarian development and function (Bentley et al., 2010). However, few studies have examined the regulatory effects of GnIH on steroid hormone secretion and related pathways of granular cells as well as their underlying mechanisms.

The aim of our study was to investigate (1) changes in the secretion of important steroid hormones and the expression of related genes in duck granulosa cells under treatment with different concentrations of GnIH (0, 0.1, 1, 10, and 100 ng/mL, duck species GnIH: SIKPIANMPLRF) over 24 h and (2) novel genes that regulate the steroid synthesis pathway by RNA-seq in the 0 and 10 ng/mL GnIH treatments.

## Methods

### Isolation and culture of granulosa cells

Small yellow follicles (SYFs, 5–12 mm in diameter) were collected immediately from the ovaries of ten laying ducks and placed in ice-cold phosphate-buffered saline (PBS, Gibco, Grand Island NY, USA) with 100 U/mL of penicillin or streptomycin (Gibco, USA). The granulosa cells were carefully separated from SYFs and treated with collagenase II (Biosharp, China) at 37 °C for 20 min. An equal volume of M199 medium (M199, Gibco, USA) containing fetal bovine serum (FBS, Gibco, South America) was added to stop the digestion, and the cell suspension was filtered through a 70-μm cell strainer (JET BIOFIL). After centrifuging the filtrate at 1,000 rpm for 5 min at 4 °C, it was resuspended in M199 medium containing FBS and 100 U/mL of penicillin or streptomycin. Cell viability and concentration were assessed by an automatic cell counter (BodBoge, Shenzhen, China). A total of 10 μL of the cell suspension was introduced onto the counting slide, and the live cell count and percentage were automatically measured. Next, the cells were plated at a density of 1×10^6^/mL cells in 6-well plates (Corning Costar) and cultured with 5% CO_2_ at 39 °C. When cells had reached confluence, the medium was replaced with M199 containing different concentrations of GnIH (0, 0.1, 1, 10, and 100 ng/mL) for 24 h. The cells were also plated at a density of 5×10^7^ cells in 10-cm diameter Petri dishes (Corning Costar, NY, USA) and cultured with 5% CO_2_ at 39 °C. The cells were treated with different concentrations of GnIH (0 and 10 ng/mL) for 24 h and collected for transcriptome sequencing.

The culture medium collected from the 6-well plates was used to measure steroid hormone (E1, T, E2, P4, ACV, and INH) secretion levels by sandwich method ELISA, and the cells were used to measure the expression levels of eight key genes (*STAR*, *CYP11A1*, *CYP17A1*, *CYP19A1*, *3-β-HSD*, *FSHR*, *LHR*, and *GnIHR*) by qPCR.

### RNA extraction, library preparation, and RNA sequencing

Total RNA was extracted from granulosa cells using Trizol reagent (Invitrogen, Carlsbad, CA, USA) following the manufacturer’s recommendations. RNA-seq transcriptome libraries were prepared with the TruSeq RNA Sample Preparation Kit from Illumina (San Diego, CA, USA) using 1 μg of total RNA. Messenger RNA was then isolated by polyA selection using oligo (dT) beads and fragmented using fragmentation buffer. cDNA synthesis, end-repair, A-base addition, and ligation of the Illumina-indexed adaptors were performed per the manufacturer’s protocol. Libraries were then size-selected for cDNA target fragments of 200–300 bp on 2% Low Range Ultra Agarose followed by 15 cycles of PCR amplification using Phusion DNA polymerase (M0530L, NEB). The PCR reaction mixture (50 μL) contained 25 μL of HIFI Amplification Mix, 2 μL PCR Primer Mix, and 23 μL target fragments. The reaction procedure was as follows: 98 ºC for 45 s; followed by 15 cycles of 98 ºC for 15 s, 60 ºC for 30 s and 72 ºC for 30 s; 72 ºC for 5 min. Paired-end (2×151 bp read length) libraries were sequenced using a Truseq SBS Kit (300 cycles, Illumina).

### Read quality control and mapping

The raw paired-end reads were trimmed and quality controlled by Trimmomatic (Bolger et al., 2014) with the following parameters: ILLUMINACLIP: TruSeq2-PE.fa:2:30:10 LEADING:3 TRAILING:3 SLIDINGWINDOW:4:15 MINLEN:75. The clean reads were then aligned to the reference genome (Huang et al., 2013) *via* HIAST2 (version 2.0.5) (Kim et al., 2015a) software using the default settings.

### Analysis and annotation of DEGs

SAM files were sorted using the sort procedure of SAMtools (version 1.7) (Li et al., 2009). Using the program htseq-count (version 0.11.2) (Anders et al., 2015), the sorted SAM files were used to generate read counts expressed per gene per library to calculate the expression level of each transcript using fragments per kilobase of exon per million mapped reads (FPKM) (Roberts et al., 2011), as well as conduct DEGs analysis using functions within the R package NOIseq (version 2.22.1) (Tarazona et al., 2011). Genes were considered differentially expressed if the probability was ≥0.8 and fold-change was ≥1.5. GO functional enrichment analysis was performed using the R package GOSeq (version 1.10.0) (Young et al., 2010) and topGO (version 2.10.0), and Kyoto Encyclopedia of Genes and Genomes (KEGG) pathway analysis was performed using KOBAS (version 2.0.12) (Xie et al., 2011). DEGs were considered significantly enriched in GO terms and metabolic pathways when their Bonferroni-corrected *P*-value was ≤0.05.

### qPCR analysis

The design of the primer sequences was based on Primer 5.0 software and synthesized by Sangon Biotech Co., Ltd. (Shanghai, China) (Table 1). qPCR was performed using an ABI7500 Real-Time PCR system (Applied Biosystems, Foster City, CA, USA). Reactions were performed using the SYBR Prime Script RT-PCR Kit (Takara Bio Inc, DRR041A, Japan) per the manufacturer’s instructions. Melt curve analysis and efficiency tests for primers were carried out to ensure primers were amplified as a single product with 90–110% efficiency. The qPCR reaction mixture (20 μL) contained 1 μL of sample cDNA, 0.2 μM forward and reverse primers, 8.6 μL of DNase-free water, and 10 μL of SYBR Premix Dimer Eraser. The reaction procedure was as follows: 95 ºC for 30 s, followed by 40 cycles of 94 ºC for 5 s and 60 ºC for 30 s. The fold-changes in gene expression levels were calculated using the 2^-△△CT^ method (Livak and Schmittgen, 2001). The glyceraldehyde-3-phosphate dehydrogenase gene (*GAPDH*) was used as the reference gene.

**Table 1:** qPCR primer sequences.

**Gene**	**Primer sequence (5′–3′)**	**NCBI reference sequence**
*GAPDH*	F:GCTGATGCTCCCATGTTCGTGAT	XM_027449739.1
	R:GTGGTGCAAGAGGCATTGCTGAC	
*FSHR*	F: AGTGTTGAATGTACTCGCCT	XM_021267212.2
	R: TGTCTGTTCTGTAAATCTGG	
*LHR*	F: GCTCTGTGATAACTTGCGTA	EU049613.1
	R: TGAGGTTTCTGTTGTCCTTC	
*STAR*	F: CTGCCATCTCCTACCGCCAC	XM_027443533.1
	R: CTGCTCCACCACCACCTCCA	
*CYP11A1*	F: ACAGGGAGAAGTTGGGTGTC	KY463321.1
	R: GTAGGGCTTGTTGCGGTAGT	
*CYP17A1*	F: GTCGCCCTGGAGAAGATCAT	NM_001001901.2
	R: TCGGTAGGAGGAGTTGAAGC	
*3-β-HSD*	F: AGAAGTGACAGGCCCAAACT	KC310447.1
	R: ACATGGATCTCAGGGCACAA	
*CYP19A1*	F: GGATGGGAGTAGGTAATGCC	KY762997.1
	R: ACAAGACCAGGACCAGACAG	
*GnIHR*	F: CATCCTGGTGTGCTTCATCG	KC514473.1
	R: ACATGGTGTTGTCAAAGGGC	

### Statistical analysis

All results were presented as the means ± standard error of the mean (SEM) from at least three independent experiments, each one performed in triplicate. Results were analyzed using one-way ANOVA and multiple comparisons by GraphPad Prism 7 (GraphPad Software, La Jolla, CA, USA). The threshold for significance was *P*< 0.05.

### Ethics statement

All experimental procedures in this study were performed in accordance with the Regulations of the Administration of Affairs Concerning Experimental Animals (State Science and Technology Commission in China, 1988) and EU Directive 2010/63/EU on the use of animals for research. All animal experiments were approved by the Animal Care and Use Committee of Zhongkai University of Agriculture and Engineering (Permit Number: 2020-014).

## Results

### GnIH treatment downregulated steroid hormone secretion and gene expression in granulosa cells

**Figure 1:**
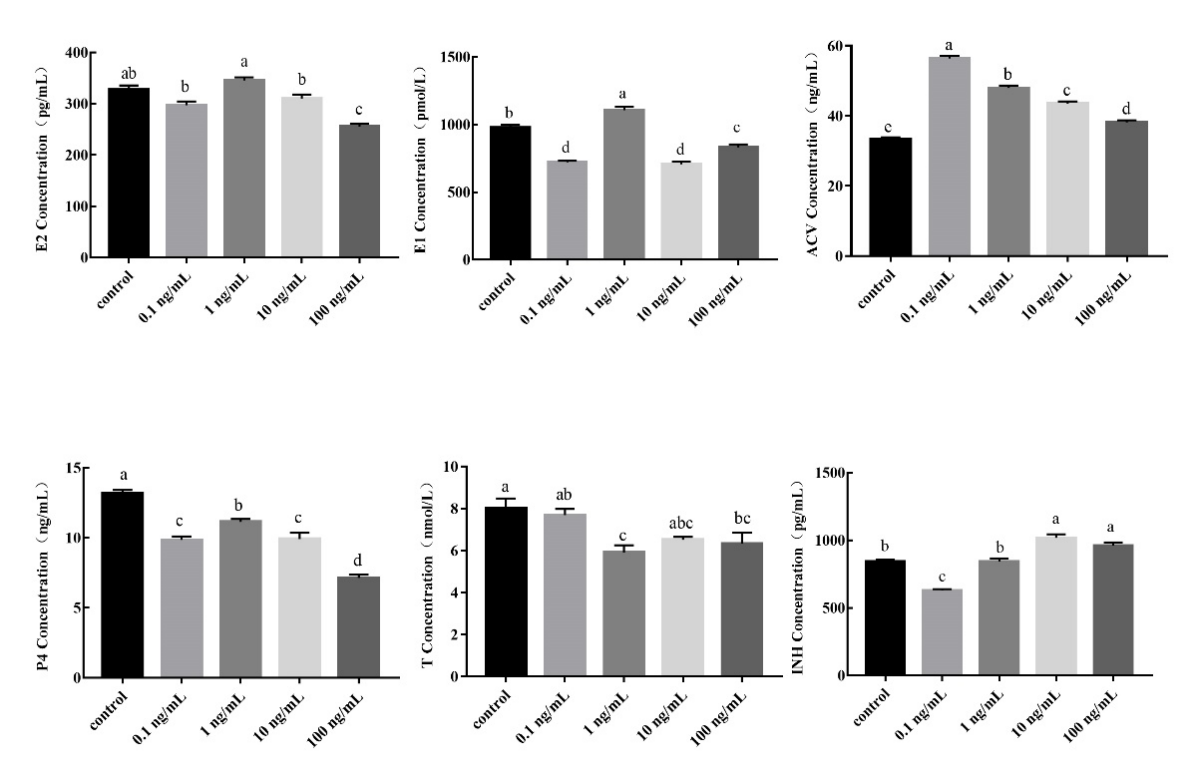
Effect of GnIH treatment on E2, E1, P4, T, ACV, and INH secretion levels. Values were expressed as means ± SEM. Different lowercase letters indicate significant difference (*P*<0.05); The inclusion of the same lowercase letters indicate no significant difference (*P*>0.05).

We treated duck granulosa cells with different concentrations of GnIH (0, 0.1, 1, 10, and 100 ng/mL) for 24 h and then measured the levels of secreted P4, E1, E2, T, ACV, and INH in the culture medium. The levels of E1 and P4 secretion were lower (*P*<0.05) under high GnIH concentrations (10 and 100 ng/mL) than in the control group (0 ng/mL GnIH) (Figure 1). Treatment with higher GnIH concentrations also inhibited the amount of E2 and T secreted by the granulosa cells; however, treatment with 10 ng/mL GnIH had no significant effect (*P*>0.05). Compared with the control group (0 ng/mL GnIH), the levels of secreted P4 and T were significantly reduced (*P*<0.05) in the 1 ng/mL GnIH treatment; the levels of secreted INH and ACV were significantly increased (*P*<0.05) in the 10 and 100 ng/mL GnIH treatments.

**Figure 2:**
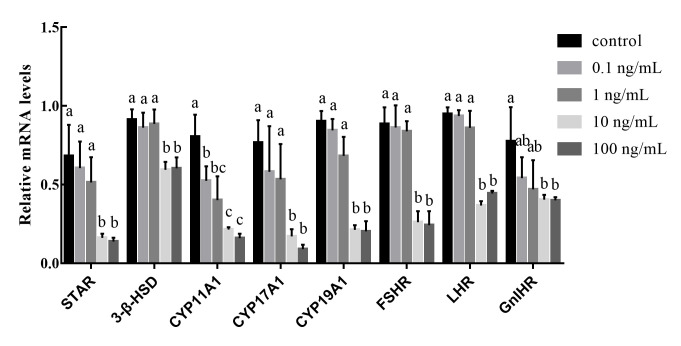
Effect of GnIH treatment on the expression of reproduction related genes. Gene expression was determined by qPCR. The results were normalized based on the *GAPDH* housekeeping gene. All values were expressed as the mean ± SEM. Different lowercase letters indicate significant difference (*P*<0.05); The inclusion of the same lowercase letters indicate no significant difference (*P*>0.05).

qPCR was performed to validate reproduction-associated gene expression in duck granulosa cells treated with different concentrations of GnIH (0, 0.1, 1, 10, and 100 ng/mL). The expression of *STAR*, *3-β-HSD*, *CYP11A1*, *CYP17A1*, *CYP19A1*, *FSHR*, *LHR*, and *GnIHR* was downregulated in the 0.1 and 1 ng/mL GnIH treatment groups compared with the control group (0 ng/mL GnIH) (Figure 2); however, only the expression of *CYP11A1* reached statistical significance (*P*<0.05). The mRNA expression levels of these genes were all significantly downregulated (*P*<0.05) in cells treated with higher GnIH concentrations (10 and 100 ng/mL) relative to cells in the control group (0 ng/mL GnIH).

### RNA sequencing

**Figure 3:**
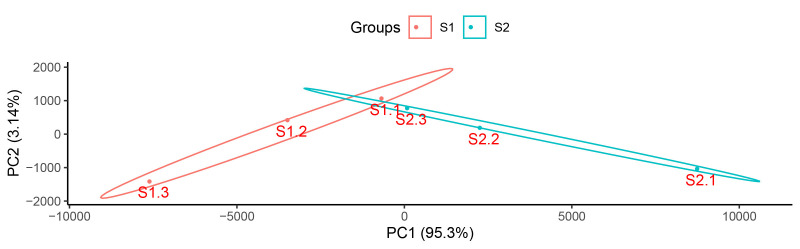
Principal component analysis scatterplot of samples.

Granulosa cells treated with different concentrations of GnIH (0 and 10 ng/mL) for 24 h were used for high-throughput RNA sequencing, and there were 3 replicates for each treatment. We obtained 36.87 G of clean bases for 6 samples. Quality evaluation of the raw sequencing data showed that the Q20 ratio of clean reads for all samples exceeded 97%, and the genomic alignment rate was over 77% (Table 2). Principal component analysis (PCA) showed that the gene expression patterns in the 10 ng/mL GnIH treatment group were markedly different from those of the control group (0 ng/mL GnIH) (Figure 3).

**Table 2:** Clean reads statistics.

**Sample**	**Total Clean Reads**	**Total Mapping Ratio**	**Uniquely Mapping Ratio**	**Q20 (%)**	**Q30 (%)**
S1-1	52615692	78.21%	71.62%	98.24	94.26
S1-2	50247302	78.49%	72.07%	98.03	93.71
S1-3	43186154	80.51%	74.07%	98.13	93.93
S2-1	36420012	77.04%	69.58%	97.92	93.49
S2-2	48833142	77.71%	70.90%	97.9	93.45

### DEGs in duck granulosa cells

**Figure 4:**
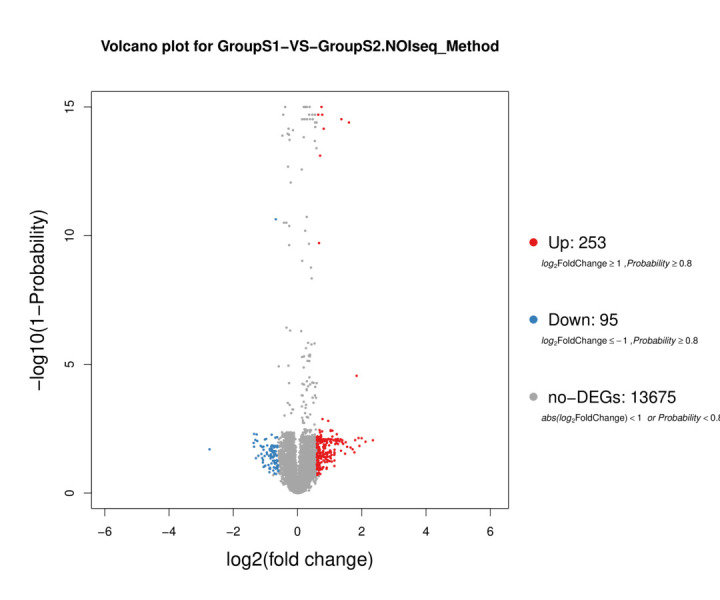
Volcano plot of DEGs in duck granulosa cells. Each dot represents a gene. Red dots indicate upregulated genes, and blue dots indicate downregulated genes. Grey dots indicate genes that were not differentially expressed.

A total of 13,794 expressed genes were detected, and 348 DEGs were identified, including 253 upregulated and 95 downregulated genes (Figure 4). The top 10 upregulated genes were *LOC101790824*, *EGR1*, *FDXR*, *IL8*, *SLC15A1*, *LOC101801765*, *ATP1B1*, *CLIC6*, *LRRC52*, and *GAS1*. The top 10 downregulated genes were *HAS2*, *LAMA3*, *CDCA7*, *LOC113842153*, *ESRRB*, *ISM1*, *PRICKLE1*, *FHOD3*, *LOC113840872*, and *ARHGAP20*. The ID, description, and summary of the functions of these genes are shown in Table 3 and Table 4.

**Table 3:** The top 10 upregulated genes in granulosa cells.

**Gene ID**	**Gene Name**	**Description and Summary of Function**
XM_005026649.4	*LOC101790824*	
XM_027468454.1	*EGR1*	A nuclear protein that functions as a transcriptional regulator
XM_027471381.1	*FDXR*	Encodes a mitochondrial flavoprotein, initiates electron transport for cytochrome P450, receives electrons from NADPH
NM_001310420.1	*IL8*	A major mediator of the inflammatory response
NM_001310803.1	*SLC15A1*	Encodes an intestinal hydrogen peptide cotransporter and is important for the uptake and digestion of dietary proteins
XM_027454493.1	*LOC101801765*	
NM_001310386.1	*ATP1B1*	Encodes an integral membrane protein, maintains an essential electrochemical gradient
XM_027449142.1	*CLIC6*	Encodes chloride intracellular channel family of proteins
XM_027462893.1	*LRRC52*	An auxiliary protein of the large-conductance, voltage- and calcium-activated potassium channel
XM_027446434.1	*GAS1*	Plays a role in growth suppression, blocks entry into S phase, and prevents cell cycling

**Table 4:** The top 10 downregulated genes in granulosa cells.

**Gene ID**	**Gene Name**	**Description and Summary of Function**
XM_005019040.4	*HAS2*	Related to glycosaminoglycan metabolism and metabolism
XM_027451936.1	*LAMA3*	Related to nanoparticle effects and cell junction organisation
XM_027461168.1	*CDCA7*	Related to validated targets of C-MYC transcriptional activation
XM_027448834.1	*LOC113842153*	
XM_027459472.1	*ESRRB*	Related to the Wnt signalling pathway and Pluripotency
XM_027453339.1	*ISM1*	Acts as an angiogenesis inhibitor
XM_027457011.1	*PRICKLE1*	Related to Wnt signalling pathways: CDK-mediated phosphorylation and removal of Cdc6
XM_021276453.2	*FHOD3*	Related to binding and actin binding
XM_027447614.1	*LOC113840872*	
XM_027468631.1	*ARHGAP20*	Related to signaling by GPCRs and Signalling by Rho GTPases

### Gene Ontology (GO) and KEGG analysis

**Figure 5:**
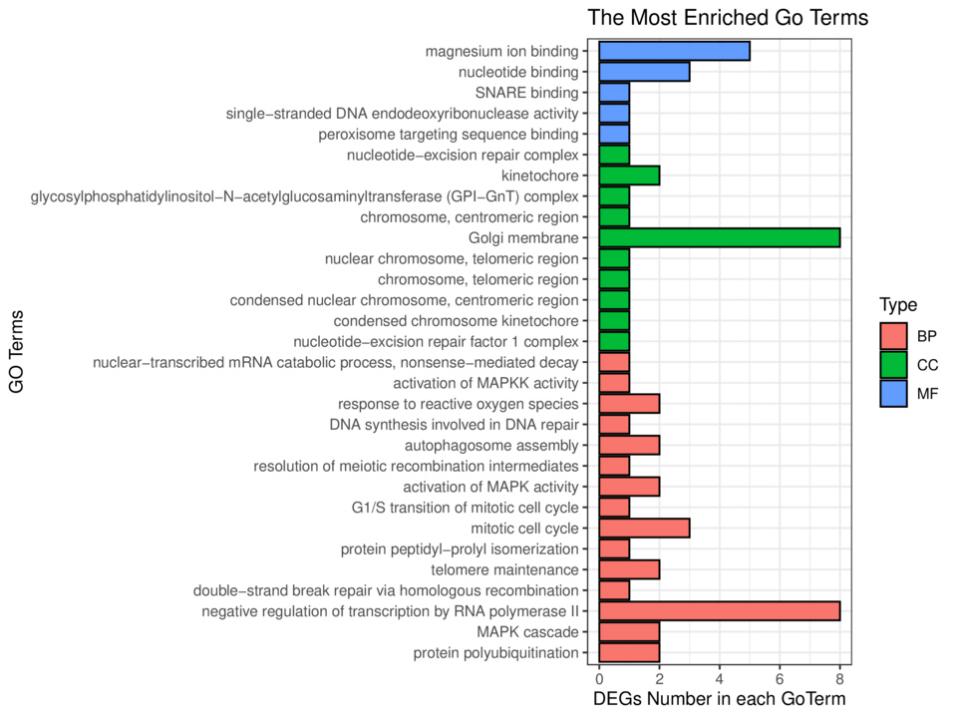
Histogram of Gene Ontology (GO) Terms. Different colors were used to distinguish biological process (BP), cellular component (CC), and molecular function (MF). Blue represents MF, green represents CC, and orange represents BP.

**Figure 6:**
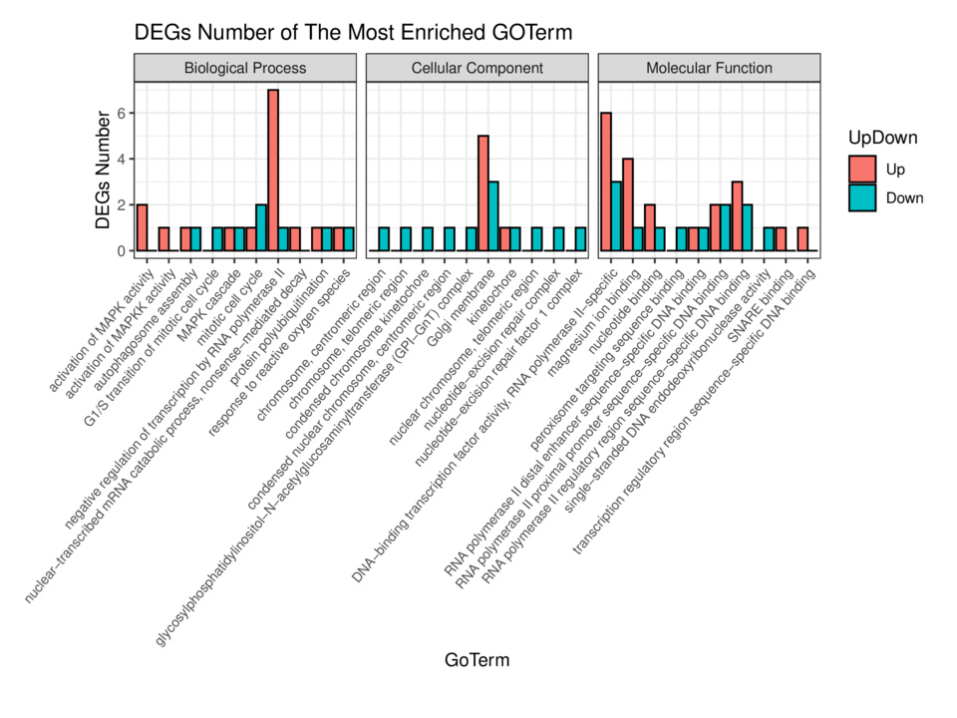
Histogram of enriched GO Terms for DEGs. The orange represents upregulated genes, and green represents downregulated genes. The *x*-axis and *y*-axis indicate the enriched GO Terms and the number of DEGs, respectively.

**Figure 7:**
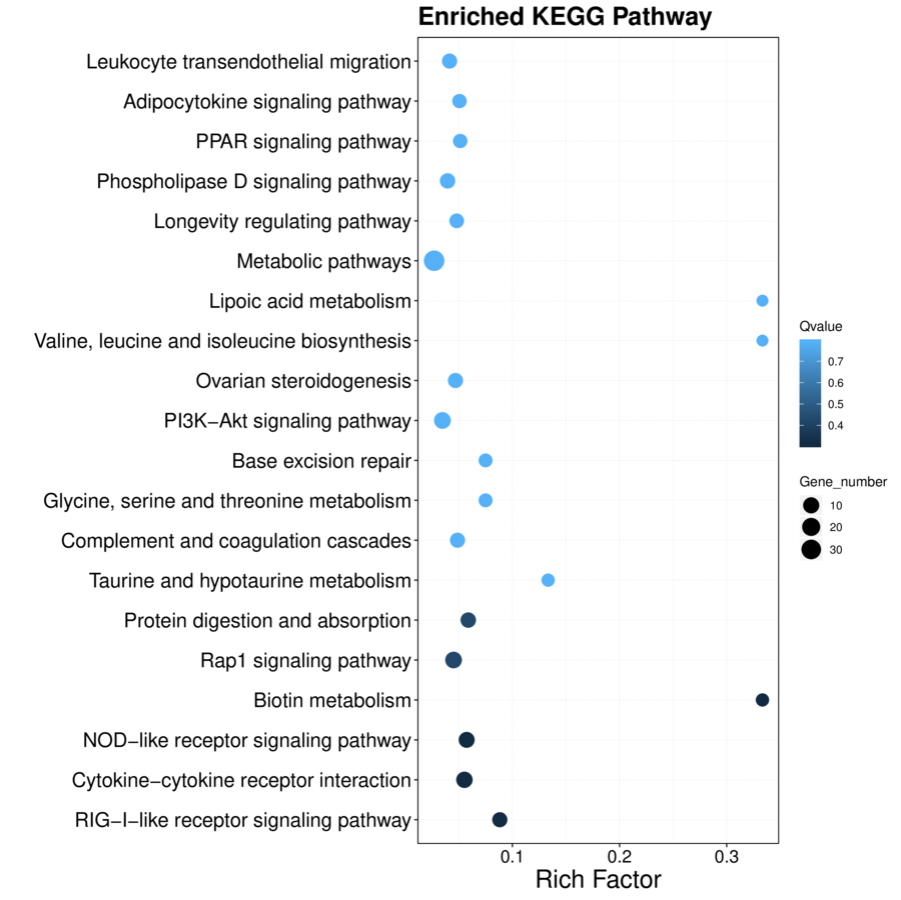
Scatter plot of enriched pathways for DEGs in granulosa cells. The *x*-axis and *y*-axis indicate the rich factor and pathway entry, respectively. The size of the dots indicates the number of significant DEGs; larger dots correspond to a greater number of significant DEGs. Different colored points represent different *Q*-values.

GO enrichment analysis was performed, and the top 30 significantly enriched GO Terms were examined (Figure 5). The most significantly enriched GO terms were enriched in magnesium ion binding in the molecular function (MF) category, Golgi membrane in the cellular component (CC) category, and mitotic cell cycle in the biological process (BP) category. To explore whether the GO Term functions were dominated by upregulated or downregulated genes, we selected the top 10 significantly enriched GO Terms from BP, CC, and MF. The upregulated genes were mainly enriched in the negative regulation of transcription by RNA polymerase II, and the downregulated genes were mainly enriched in Golgi membrane (Figure 6). According to the KEGG enriched analysis, the DEGs were mainly enriched in 20 key pathways, such as RIG-I-like receptor signalling pathway, Cytokine–cytokine receptor interaction, and Ovarian steroidogenesis (Figure 7).

**Figure 8:**
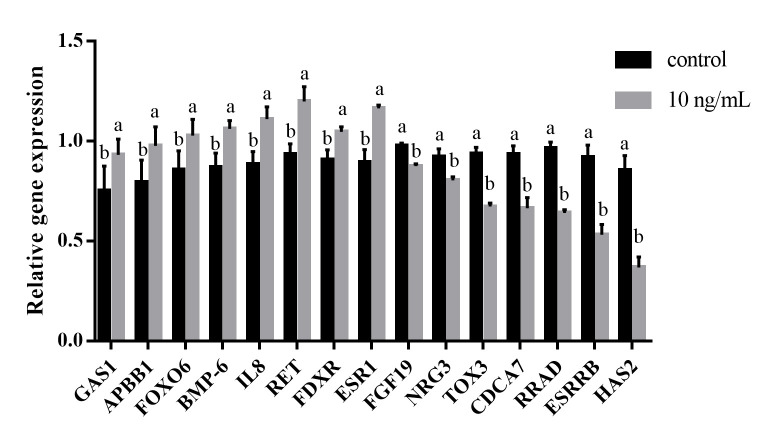
qPCR validation of key DEGs in granulosa cells. Gene expression levels were assessed by qPCR and were presented relative to *GAPDH* expression. Statistical significance was assessed by *t*-test. Different lowercase letters indicate significant difference (*P*<0.05).

The 15 key DEGs were selected for validation using qPCR, including MAPK pathway-activator genes (*FGF19* and *RET*); cell growth, apoptosis, and cell cycle-related genes (*APBB1*, *GAS1*, *RRAD*, *FOXO6*, *NRG3*, *TOX3*, *CDCA7*); steroid biosynthetic process-related genes (*ESR1*, *ESRRB*); the immune factor *IL8*; and three other genes (*HAS2*, *FDXR*, *BMP-6*). The verification results for the expression of the DEGs were consistent with those for transcriptome sequencing, indicating that the sequencing results were reliable (Figure 8).

### Steroidogenesis pathway analysis

**Figure 9:**
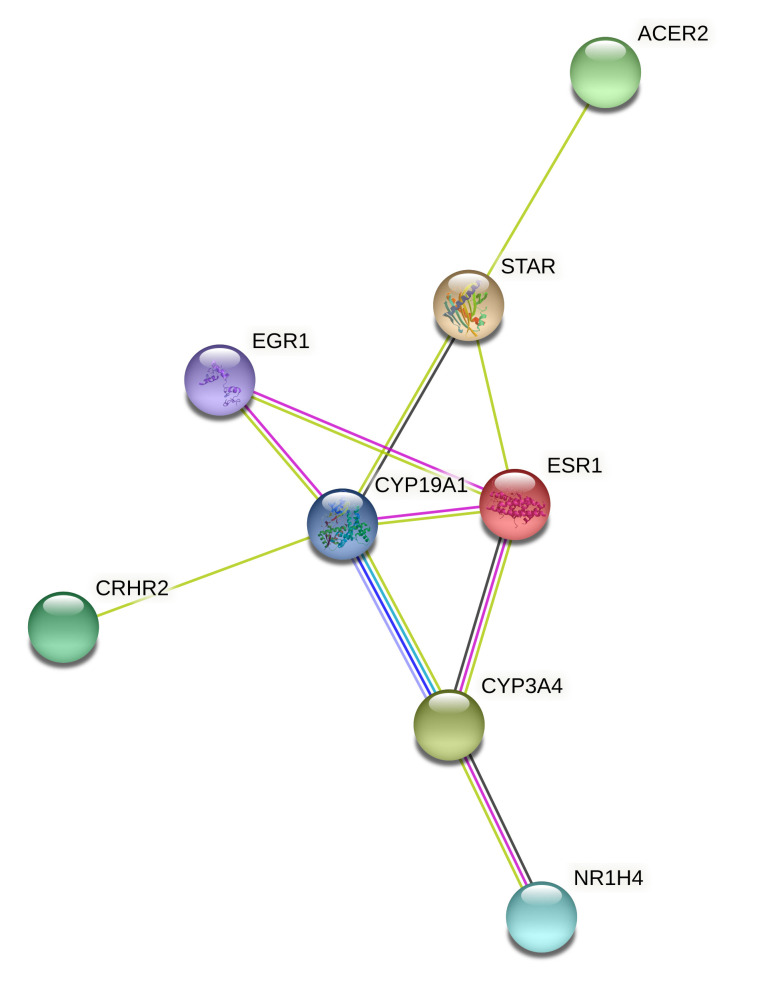
Protein–protein interaction (PPI) network of eight DEGs based on the STRING database.

KEGG pathways functional analysis found that six DEGs were enriched in the ovarian steroidogenesis pathway, including *MROH5*, *LOC113840576*, *SDR42E1*, *LOC113841457*, *STAR*, and *CYP19A1*. Four novel genes, *MROH5*, *LOC113840576*, *SDR42E1*, and *LOC113841457*, were first reported to be related to the ovarian steroidogenesis pathway. We also used these eight DEGs to construct a protein–protein interaction (PPI) network based on the STRING database. Six other genes (*EGR1*, *ESR1*, *ACER2*, *CRHR2*, *CYP3A4*, and *NR1H4*) interacting with DEGs were involved in the pathways of steroid hormone biosynthesis (Figure 9). These genes were found among DEGs and are potential candidate genes for understanding the molecular mechanism underlying reproduction in ducks.

## Discussion

The recently identified hypothalamic neuropeptide, GnIH, is a key regulator of reproduction. The peripheral injection of GnIH has been shown to inhibit FSH and LH synthesis and release in chicken (Maddineni et al., 2008; McGuire et al., 2011). In mammals, GnIH receptors are distributed in porcine ovarian granulosa cells and reduce the levels of the proliferation-related proteins ERK1/2, cyclin-B1, and PCNA after GnIH treatment (Li et al., 2013). Additionally, GnIH treatment can reduce ovarian activity and inhibit follicular development in mice (Anjum et al., 2012; Singh et al., 2011a). These findings indicate that GnIH affects the proliferation and development of follicular granulosa cells.

We assessed the expression of *STAR*, *3-β-HSD*, *CYP11A1*, *CYP17A1*, and *CYP19A1*, the marker genes of steroid synthesis in granulosa cells, after different concentrations of GnIH treatment. These steroid synthesis marker genes play a crucial role in follicular development and the steady state of steroid hormones (Guo et al., 2019). qPCR analyses showed that the expression levels of these genes were downregulated in granulosa cells after GnIH treatment. Previous research has also shown that GnIH treatment dose-dependently suppressed steroidogenesis by inhibiting the gene expression of *STAR* and *3-β-HSD* in the ovary of mice (Singh et al., 2011b). Moreover, GnIH treatment also decreased the expression of *FSHR*, *LHR*, and *GnIHR*. These findings indicate that GnIH regulates the proliferation of granulosa cells by reducing the expression of steroid synthesis-associated genes and related receptors. Steroid hormone secretion is essential for the reproduction and development of animals. Steroid synthases catalyze the synthesis of multiple hormones, including T, P4, E2, and E1 (Payne and Hales, 2004). Our results showed that GnIH treatment reduced the secretion level of these steroid hormones. The reduction in the level of P4 may be related to the decreased *STAR* and *CYP11A1* mRNA expression levels (Mosa et al., 2015). Previous studies have also indicated that GnIH treatment reduces the secretion levels of E2 and P4 (Li et al., 2013). Based on these findings, we hypothesized that the downregulated expression of *ESRRB* and *CYP19A1* may inhibit estrogen biosynthesis in granulosa cells, and this in turn results in a low E2 level. The secretion of E2 and P4 has also been confirmed to affect the expression of *GnIHR* (Maddineni et al., 2008). The lack of E2 also leads to granulosa cell apoptosis, which is consistent with the downregulation of apoptotic gene expression (Shimizu et al., 2005). The secretion level of P4 and the expression of *3-β-HSD* were both significantly decreased, indicating that GnIH had a direct inhibitory effect on follicular development. In addition, the increase in INH expression may have helped maintain androgen synthesis in theca cells (Johnson, 1993). The upregulation of INH and ACV also had important effects on the proliferation and development of granulosa cells (McNatty et al., 2000; Lovell et al., 2003). In this study, the expression of reproduction-associated genes was downregulated, which was consistent with the pattern of steroid hormone secretion. These results indicate that GnIH regulates the secretion of steroid hormones and affects the reproduction and development of granulosa cells.

In addition, we further studied the effect of GnIH on granulosa cells by high-throughput sequencing. A total of 348 significant DEGs were detected, which were mainly significantly enriched in pathways such as Rap1 signaling pathway, Cytokine–cytokine receptor interaction, PI3K-Akt signaling pathway, and Ovarian steroidogenesis. Many of the significantly enriched pathways were related to the immune system and the reproductive and endocrine systems. In addition, the expression of numerous genes associated with cell cycle, cell division, and DNA replication was consistent with the physiology of proliferation and development of granulosa cells (Knight and Glister, 2006; Zhu et al., 2019). These signaling pathways may control ovarian follicular and ovulation development. Both chicken and bovine follicles showed a similar expression profile in steroidogenesis adjustment in follicular development (Hatzirodos et al., 2014).

We then selected specific DEGs to validate their levels of expression. GnIH treatment led to a significant increase in *IL8* gene expression and affected multiple immune-related pathways, indicating that GnIH is involved in the immune response in granulosa cells. Previous studies have also shown that GnIH immune reactive neurons were widely distributed in thymus, medulla and lymph nodes, thereby modulating a variety of immune functions (Wang et al., 2018). This may be achieved by regulating *IL8* gene expression. In terms of reproduction regulation, the expression of *FOXO6*, *APBB1*, and *GAS1* was upregulated after GnIH treatment, whereas that of *NRG3*, *TOX3*, *CDCA7*, and *RRAD* was downregulated. *GAS1* plays a role in growth suppression, and *CDCA7* is involved in MYC-mediated cell transformation and apoptosis. These changes in gene expression levels indicate that GnIH affects the cell cycle, growth, and apoptosis of granulosa cells. Previous studies have also demonstrated that GnIH affects the cell cycle and induces cell cycle arrest at the G2/M phase in porcine ovarian granulosa cells (Wang et al., 2018). GnIH plays a regulatory role in mediating epididymal cell apoptosis and autophagy during reproduction in male rats (Wang et al., 2018). Notably, BMPs belong to the TGF-beta ligand superfamily and are key players in follicular development (Reddi and Reddi, 2009). BMP6 has been reported to affect granulosa cell proliferation (Ocon-Grove et al., 2012). Our results showed that GnIH treatment leads to an increase in the expression of *BMP6*, suggesting that it may regulate the proliferation of granulosa cells and follicular development through the TGF-β signaling pathway. GnIH also modulates the expression of *FGF19* and *RET* in granulosa cells, suggesting that GnIH may be involved in regulating the apoptosis of granulosa cells and the synthesis of intracellular gonadotropins by the MAPK signaling pathway (Tamura et al., 2004). GnIH treatment also affects the expression of *ESR1* and *ESRRB* in the process of estrogen receptor activity and steroid binding. *ESRRB* is specifically expressed during follicular development (E et al., 2019). Downregulation of *ESRRB* expression revealed that GnIH treatment reduces steroid hormone receptor activity. These results indicated that GnIH affects the reproduction and development of granulosa cells by regulating associated signaling pathways and the expression of genes.

KEGG pathway functional analysis indicated that *MROH5*, *LOC113840576*, *SDR42E1*, *LOC113841457*, *STAR*, and *CYP19A1* were enriched in the ovarian steroidogenesis pathway. The PPI network shows that six other genes (*EGR1*, *ESR1*, *ACER2*, *CRHR2*, *CYP3A4*, and *NR1H4*) interacting with DEGs were involved in the pathways of steroid hormone biosynthesis. We found that the top interacting DEGs were *CD44*, *ESR1*, *KITLG*, *NGFR*, *IL8*, *PTX3*, *CYP19A1*, *EGR1*, and *FGF19*. These DEG interactions were mainly enriched in Cell growth and death, Ovarian steroidogenesis, Immune system, and Endocrine system. Overall, our data provide new insight into the role of GnIH in regulating the proliferation and development-related pathways of duck granulosa cells.

## Conclusion

Our study showed that GnIH treatment reduced the secretion of steroid hormones and the expression of reproduction-associated genes in duck granulosa cells in a concentration-dependent manner. Transcriptome analysis of duck granulosa cells treated with GnIH (0 and 10 ng/mL) identified several DEGs that may be involved in immune responses, cell growth, and steroid biosynthesis pathways. We identified four novel DEGs (*MROH5*, *LOC113840576*, *SDR42E1*, *LOC113841457*) that play important roles in the regulation of the steroid synthesis pathway. This study provided new insight into the inhibitory effects of GnIH on steroid synthesis in duck granulosa cells and identified several genes that may be involved in this process. More generally, the results of this study improve our understanding of the molecular mechanisms underlying duck reproduction.
